# Simple manual roller pump-driven valve-free microfluidic solution exchange system for urgent bioassay†

**DOI:** 10.1039/d1ra08052k

**Published:** 2022-01-21

**Authors:** Gokul Chandra Biswas, Hiroaki Suzuki

**Affiliations:** School of Life Sciences, Shahjalal University of Science and Technology Sylhet-3114 Bangladesh gcbiswas-geb@sust.edu; Graduate School of Pure and Applied Sciences, University of Tsukuba 1-1-1 Tennodai Tsukuba Ibaraki 305-8573 Japan

## Abstract

We introduce a simple-to-use manual roller pump (MRP)-driven and valve-free microfluidic system for sequential solution exchange, followed by a bioassay to detect protein. The polydimethylsiloxane (PDMS)/glass-based disposable device comprises a reaction chamber, multiple micro-flow channels (μFCs), and air vents. The practical solution exchange was realized by sequential injection and withdrawal of several solutions into and from the reaction chamber through constricted μFCs by utilizing changing air pressure of an MRP when a small cylindrical roller was pressed and rolled over a soft silicone tube using a finger. Furthermore, we investigated the effect of surface hydrophobicity on solution exchange. A sandwich fluorescence-based immunoassay to detect human interleukin 2 (IL-2) was performed using this simple microfluidic scheme to demonstrate its suitability for analytical bioassays. The system allowed quick IL-2 detection in 20 min in a pre-functionalized device with a detection limit of 80 pg mL^−1^ and a range of 125 pg mL^−1^ to 2.0 ng mL^−1^. We have thus developed a microfluidic scheme that non-experts can efficiently perform and that can be the fundamental module for low-cost bioassays necessary for emergencies and situations where resources are constrained.

## Abstract 2

We report an easy microfluidic solution exchange system that employs a finger-driven manual roller pump (MRP) and valveless micro-flow structures to enable minimally trained personnel to execute instantaneous stepwise bioassays.

## Graphical abstract

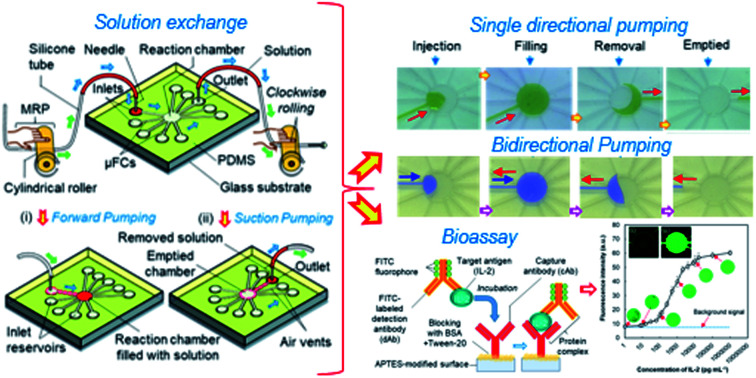

## Introduction

Infectious diseases are highly prevalent in developing countries.^1–3^ Contagious diseases can become global pandemics. They can exert catastrophic impacts on countries regardless of their developmental status, as recently exposed by the millions of infections and deaths caused by the COVID-19 pandemic.^4^ To deal with the consequences of the pandemic, the World Health Organization (WHO) urged for an increase in the quantity of diagnostic tests by promoting their “test, test, test,”^5^ and ranked point-of-care testing (POCT) as the first of eight urgent research actions identified at the COVID-19 pandemic epoch.^6^ In this respect, Micro Total Analysis Systems (μTAS)^7,8^ can act as a flagship that enables diagnosis seekers to receive low-cost, rapid diagnostic outputs from non-experts at nearby clinics, at home, or in resource-constrained settings. If realized, this method should augment personalized therapy or treatment against critical diseases in contrast to expensive, laborious, laboratory-oriented conventional diagnostic approaches.^1–3,9,10^

Alongside nucleic acid testing, protein detection can aid indirect diagnosis and prediction of immune status against particular infectious diseases.^11^ ELISA is commonly performed to detect target proteins or biomarkers;^12^ even so, traditional ELISA requires skilled operators, occupies more space and assay times, and consumes a bigger quantity of costly reagents or samples, obstructing its great chance of extensive applications for domestic or clinical diagnosis. As a problem-solving approach, microfluidics enables immunoassays on miniaturized chips or devices.^11,13,14^ The small assay chambers in micro-systems maintain the high surface-to-volume ratios to support prompt and high-throughput analysis.^13,14^ Micro-flow systems carry the enormous potential to facilitate emergency medical care with the simple analytical tool in remote corners of resource-inadequate settings. Practically, despite its prospects, the application of these systems has not been successfully expanded so far except for the lateral flow immunoassay (LFIA).^15^ This is because the simplification-allied challenges in the sequential exchange of several solutions essential for consecutive reactions and washings in microfluidic affinity biosensing is yet to be addressed.

Solution exchange for microfluidic bioassays is usually governed by microfluidic components such as pumps or valves. Among the reported pumps, pressure-driven pumps are straightforward for transporting solutions into and from the sensing/assay/reaction chamber through micro-flow channels (μFCs).^13,16,17^ However, these pumping systems rely on bulky peripheral instruments such as electric syringe pumps or pneumatic apparatus. Moreover, comparatively wide μFCs and high pressure are required to eliminate fluidic resistance.^18,19^ Otherwise, valves, such as pneumatic or check valves, are formed in μFCs to control solution delivery.^13,16,17,19–21^ To simplify pressure-driven pumping, approaches such as by a finger,^22,23^ screw,^24^ or chemicals^25^ have been reported, eliminating off-chip control for pumping. However, they involve extra fabrication steps and additional cost of a thin layer PDMS membrane, rotary electric motor, and chemical modification in the pump unit. Alternatively, pumping utilizing the passive driving forces of capillarity,^26–28^ evaporation^29^ or PDMS degassing^30^ have become attractive for bioassays; but their respective dependence on tiny capillary patterns and preset solution volumes, perpetual temperature and humidity, and more prolonged vacuum exposure generated scope of thinking more simplification regarding applications under resource constraints or in emergencies. Moreover, these micropumps may face difficulty in controlling solution flow without valve components.^27,31^ Solution exchange systems that require stopped-flow in the bioassay process may be unrealistic for these pumping approaches.

The issue can either be addressed by narrowing the μFCs in the direction of solution flow or by altering plug or column flow, either in capillary-triggered^28,31^ or syringe-driven settings.^32–34^ However, maintaining simplicity in pump operation remains problematic. Earlier, we incorporated a narrow μFC pattern on electrowetting-based valve areas and a flushing port in the reaction chamber for serial injection and removal of solutions, respectively.^35^ Later, we eased the exchange of solutions and applied wide μFCs by integrating electrochemically switchable hydrophobic valves^36,37^ and a superabsorbent pump^27^ against capillary-flowed solutions. Moreover, the solutions can be transported automatically,^37,38^ allowing this system to be operated on the spot without an external power or pressure source. However, extra fabrication steps and material costs are involved with the construction of these components.

Overall, microfluidic solution exchange without electric power and valve component remains a hurdle for on-the-spot bioassays. As a result, interest in solution pumping in microfluidic devices that does not require electricity is growing.^39–41^ Thus, it is imperative to offer structural and functional simplicity in pumping strategies and microfluidic devices to realize the practical exchange of multiple solutions and facilitate instant microfluidic immunoassays.

**Fig. 1:**
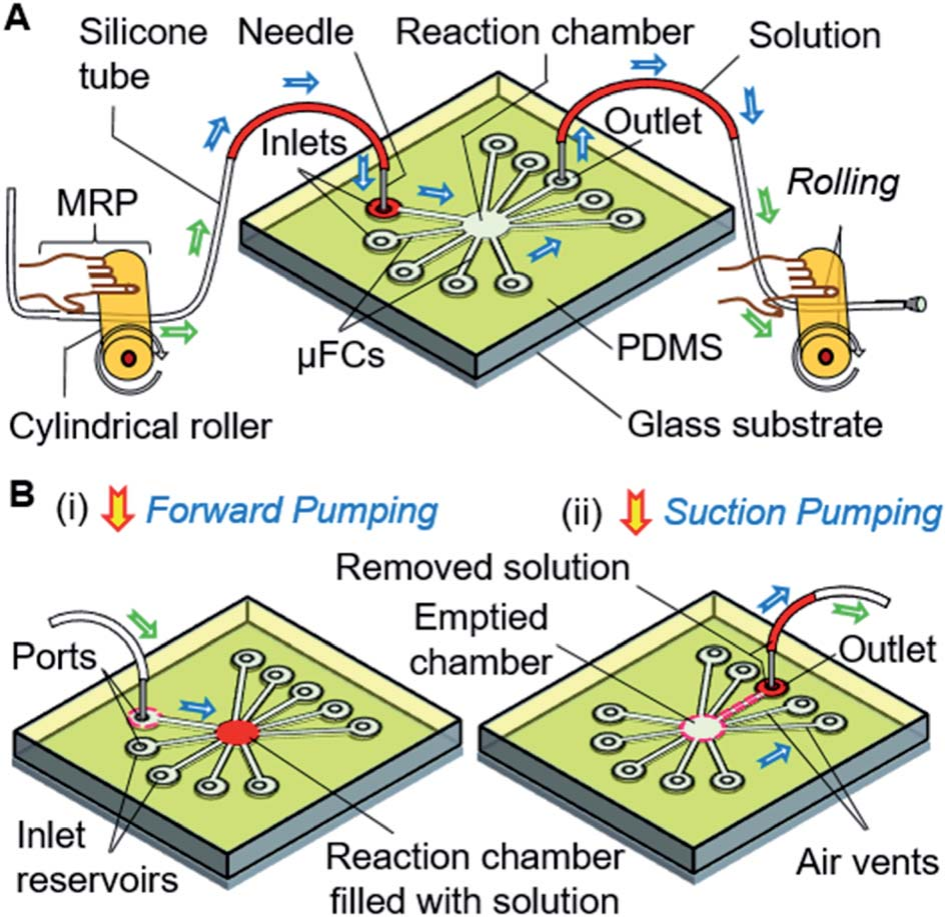
Schematic presentation of a simple microfluidic solution exchange system for bioassays. (A) A valve-free, manual roller pump (MRP)-driven microfluidic solution processing. The solution moves through the inlet and outlet tubes by utilizing air pressure generated by pumping (*i.e.*, pressing and rolling of the cylindrical roller). (B) Schematic pump operation and solution exchange. (i and ii) Subsequent solution injection into and withdrawal from the microfluidic reaction/assay chamber was realized with forward and suction pumping from the positive and negative air pressure from the MRP operation. Green and blue arrows indicate the movement of air and solution, respectively.

In this article, to address the above-stated limitations, we present a competent scheme of multiple solution exchange based on a manual roller pump (MRP)-driven valve-free simple microfluidic device. We employed an ordinary soft silicone tube with a small cylindrical roller as a pump (Fig. 1). Solution loaded in a tube or reservoir is injected and removed into and from the central reaction chamber through the connected, constricted μFCs by simple rolling of the roller with a finger, thus realizing a simple solution exchange without expensive instruments (Fig. 1A and B). Our strategy minimizes the cost and complexity of device fabrication and operation. Additionally, we demonstrated the effectiveness of the scheme by performing on-device immunoassay to detect human interleukin 2 (IL-2), which is a potential biomarker for testing/diagnosis of infections, immune response, cytotoxicity, and lymphocyte transformation.^33,42–44^ We envision this simple microfluidic approach as a valuable tool for enabling many easy-to-perform instantaneous bioassays or rapid POC tests during emergencies or in situations where access to resources is constrained.

## Experimental section

### Reagents and materials

Materials and reagents utilized for device fabrication and experiments were purchased from the following commercial sources: Glass wafers (TEMPAX Float®; diameter = 3 inches, thickness = 500 μm) from Schott Japan (Tokyo, Japan); a thick-film photoresist SU-8 25 from MicroChem (Newton, MA, USA); a negative photoresist (OMR-83) from Tokyo Ohka Kogyo (Kawasaki, Japan); a prepolymer solution of poly(dimethylsiloxane) (PDMS; KE-1300T) and (3-aminopropyl) triethoxysilane (APTES) from Shin-Etsu Chemical (Tokyo, Japan); phosphate-buffered saline (PBS, pH 7.4), bovine serum albumin (BSA), fluorescein, and a dye, sunset yellow FCF (C_16_H_10_N_2_Na_2_O_7_S_2_), from Wako Pure Chemical Industries (Osaka, Japan). Orange G (C_16_H_10_N_2_Na_2_O_7_S_2_) and methylviolet (C_24_H_28_N_3_Cl) were obtained from Merck (Darmstadt, Germany). Food dyes were obtained from Kyoritsu Foods (Tokyo, Japan). An FITC labeling kit (lot. PG201955) was procured from Thermo Scientific (Rockford, IL, USA). The standard ELISA kit for human IL-2 (Human IL-2 ELISA MAX Deluxe) was obtained from BioLegend (San Diego, CA, USA). Information regarding the concentrations of the reagents in the ELISA kit was proprietary. A PBS solution containing 0.5% BSA was used to dilute the protein solutions. A diluent in the kit was used to prepare the 200-fold diluted antibody (capture and detection antibody) solutions. All the other reagents not stated above were acquired from Wako Pure Chemical Industries (Osaka, Japan). Milli-Q pure water of 18 MΩ cm^−1^ resistivity was utilized to make all the necessary solutions.

### Design and fabrication of the device

**Fig. 2:**
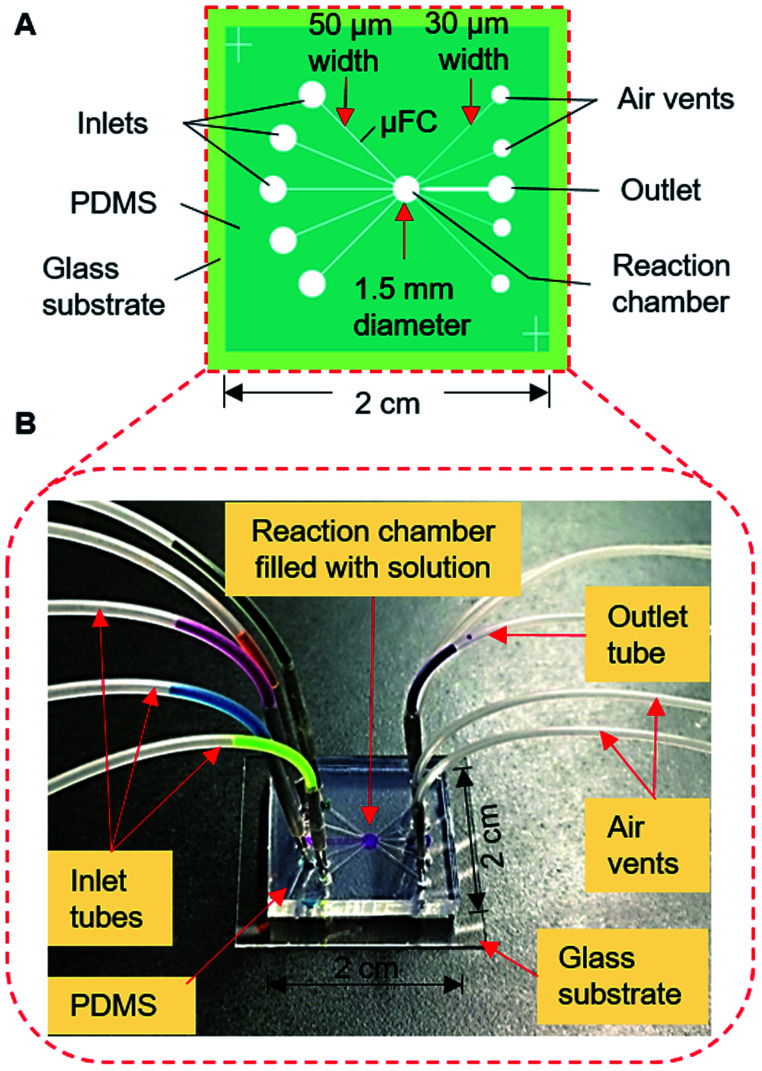
Simple valve-free microfluidic device for solution exchange in the bioassay process. (A) Schematic of the device, (B) setup for solution exchange in the fabricated device with the solution introduced and retained in the reaction chamber using the MRP.

The devices were fabricated with a PDMS substrate with microfluidic patterns and a glass substrate. A thick-film photoresist (SU-8 25) was used to form a template for the PDMS structures *via* the replica molding technique, as detailed in the ESI.†Fig. 2A shows one of the devices which was used in the final stage. Other configurations are discussed in later sections and shown in ESI (Fig. S1†). The devices contained five connecting μFCs, a reaction chamber, and four air vents in the PDMS substrate. The height of the flow channels and reaction chamber was 80 μm for all devices. The width of each of the flow channels was 50 μm. The diameter of the reaction chamber was 1.5 mm (volume: ∼140 nL). To smoothen the transport of the solution by applying air pressure, air vents of 30 μm width were added to the reaction chamber near the outlets. For injecting and removing the solution, the inlet and outlet ports were formed by making through-holes in the PDMS layer using a disposable 1 mm biopsy punch (ref. BP-10F, Lot no. 16B25; KAI Industries Co. Ltd., Seki, Japan). The outside ends of the air vents contained through-holes in the PDMS stratum. The inlets and outlet were connected to the MRP through broken needles inserted into silicone tubes (Fig. 2B). To complete the device, the patterned PDMS and glass substrate were exposed to oxygen plasma for 20 s at 20 W and 30 Pa of oxygen pressure to make strong bonding. The activated glass and PDMS substrates were afterward aligned and bonded by applying gentle pressure.

### Structure and operation of the manual roller pump

The MRP consists of a soft silicone tube (inner and outer diameters of 1 mm and 2 mm, respectively) with a connected piece of a small needle (1 mm outer diameter) inserted into the port of the inlet reservoir to load solutions and pump the device. With the assistance of a small cylindrical rigid roller, such as a glass bottle, rod, or dry cell battery, the pump is ready to use (Fig. S2†).

**Fig. 3:**
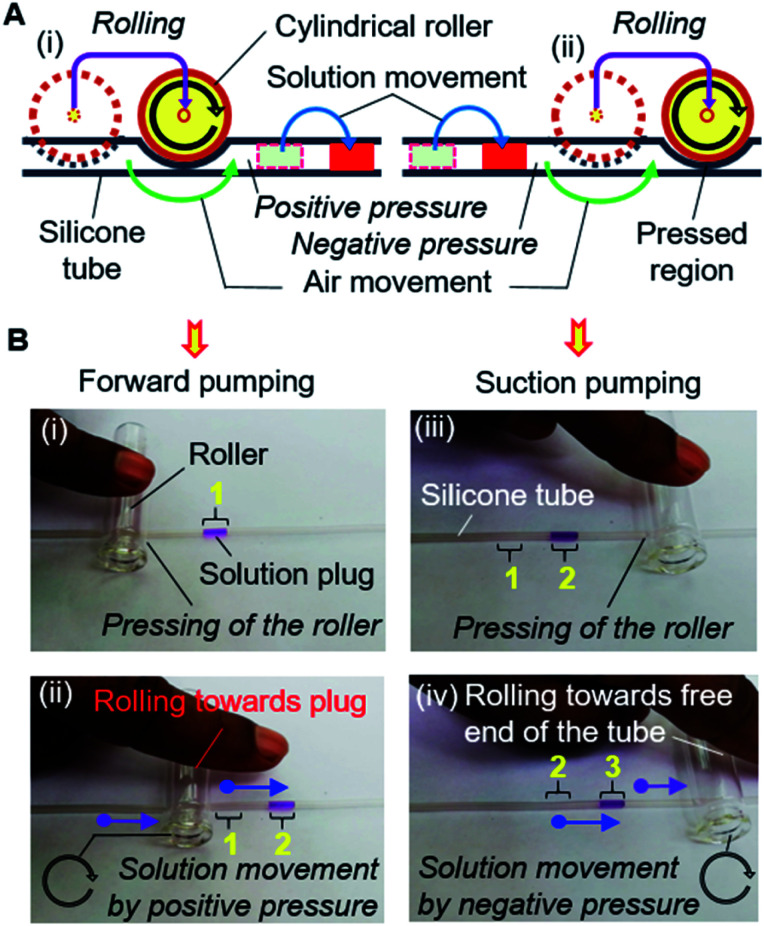
Operational principle of the manual roller pump. (A) Schematic mechanism of the MRP operation. (B) Operation of MRP for the single directional solution movement in a silicone tube by (i and ii) forward and (iii and iv) suction pumping, which employ positive and negative air pressure, respectively. Green and blue arrows show the flow of air and solution respectively; whereas, black and purple arrows point towards the rolling direction and displacement of the roller, respectively. The yellow numbers represent the position of the solution plug during operation.

The operation of the MRP is very simple and transport of a solution can be started immediately by placing, pressing, and rolling a roller on a tube (Fig. 3A). Keeping the solution plug at the front, a small roller is gently pressed using a finger so that the top and bottom surfaces of the hollow tube become fused, creating an airlock before the solution plug in the tube. Afterward, positive air pressure is generated by displacing the air towards the solution by rolling the roller over the tube, which pushes the solution plug forward to realize forward pumping, like in a pneumatic pump (Fig. 3A[i] and B[i and ii]). In another way, applying the same motion to the free end of the tube will provide a suction pump action that creates a negative air pressure in the tube, resulting in the solution moving forward by suction pumping (Fig. 3A[ii] and B[iii and iv]). We call this process roller pumping, which we applied to realize solution exchange in the microfluidic device.

### Solution exchange

Fig. 2B also shows the setup for the experiment. Different dye solutions were used to visualize the solution movements in the μFCs. First, 2–3 μL of dye solutions were loaded into each of the silicone tubes *via* 1 mL disposable syringes connected to the pumping tubes. The needle segments of the front end of the tubes were later inserted into the inlets (or solution reservoirs) of the device. To demonstrate solution exchange, the solutions were sequentially and slowly injected into the reaction chamber using forward roller pumping (Fig. 3A[i] and B[i and ii]) in the silicone tubes. After the complete filling of the reaction chamber, each of the solutions was removed *via* the outlet μFC using suction roller pumping (Fig. 3A[ii] and B[iii and iv]). Thus, a solution injection (Fig. 1B[i]) and removal (Fig. 1B[ii]) into and from a reaction chamber can be realized. The same steps were repeated to realize multiple exchanges of solutions.

Likewise, solution exchange can be performed by MRP-based air pumping with solutions in reservoirs formed in the μFC structures. In that case, the solutions are first loaded using a micropipette, and only air is pumped as described above (Fig. S3†). The volume of solutions used for analysis is determined by the volume of the reaction chamber. Therefore, as long as only the reaction chamber is filled with the solutions precisely without leakage into the other flow channels, the same volume of solutions is guaranteed and no additional procedure for the measurement of solution volumes is necessary. Then again, if the quantitative loading or delivery of a solution is necessary in the differently configured devices, a scale can be marked/printed on the outer surface of the silicone tube.

In real situations, reagent solutions used for the analysis may be loaded beforehand in the tube or solution reservoirs on the chip using any tools such as syringes or micropipettes. Sample solutions can also be loaded into the tube in the same manner. However, for this purpose, MRP can also be used without the assistance of any other tools. After loading in the tube by suction pumping, the solution plug can be moved to the solution reservoirs and reaction chamber by forward pumping.

### FITC labeling of the detection antibody

The detection IL-2 antibodies (anti-IL-2) were labeled following the instructions of Thermo Fisher Scientific. In short, 40 μL of a borate buffer solution (0.67 M) was added to 500 μL of the detection antibody (dAb) diluted to 2 mg mL^−1^ in PBS. The FITC reagent was dissolved in 500 μL of the antibody solution by pipetting the solution up and down 10 times with brief vortexing. The mixture was incubated for 60 min at 4 °C and then added to the spin column filled with a purification resin and mixed by brief vortexing to purify the labeled antibodies. The purified antibodies were collected by centrifugation of the spin column at 1000×*g* for 30–45 s. After which, the FITC-to-antibody ratio was estimated as stated by the guidelines of Thermo Fisher Scientific. Approximately four FITC fluorophores were calculated for each FITC-labeled antibody. Before labeling, all the reagents were stored at room temperature.

### On-chip fluorescence immunoassay

**Fig. 4:**
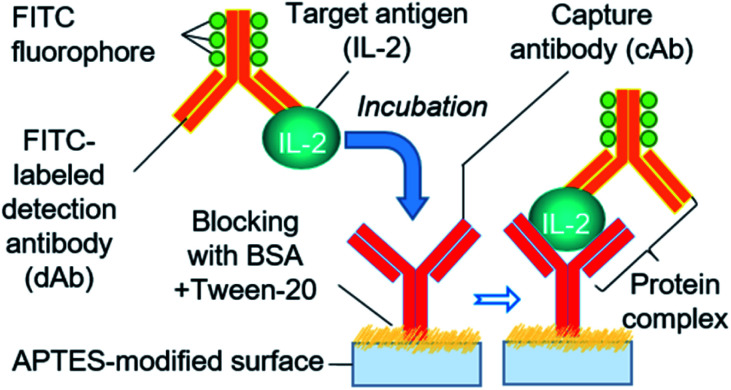
Scheme of sandwich fluorescence immunoassay for IL-2 protein detection.

We adopted a sandwich fluorescence immunoassay scheme to detect human IL-2 (Fig. 4). Unlike the traditional lengthy ELISA process, a rapid APTES functionalization-based colorimetric ELISA protocol^33^ was applied with a slight modification for fluorescence immunoassay. APTES forms a contact network for antibodies through its self-polymerization ability in an aqueous solution.^45^ Moreover, the partially polymerized APTES network offers immobilization sites for cAb *via* electrostatic interactions between the amino and carboxylic groups of APTES and cAb, respectively.^45,46^ The one-step reaction chamber functionalization and cAb immobilization started with the injection of 1 μL of instantly mixed cAb solution, APTES (1%, v/v), and coating buffer into the assay chamber, and continuing the incubation for 25 min at 4 °C. To block the non-specific binding of proteins on the surface, 1 μL of PBS containing 0.5% BSA and 0.01% Tween 20 was introduced and incubated for 30 min at room temperature after removing the previous solution and subsequent washing process. Before IL-2 detection, we formulated a standard series of IL-2 solutions by diluting the IL-2 stock solutions with PBS. To enable the rapid assay step in our system, we introduced an immediately prepared mixture of IL-2 and FITC-labeled dAb (at a 1 : 1 ratio) in the assay chamber in a single step instead of delivering the target protein and dAb solution in separate steps, as practiced in a typical immunoassay. The mixture formed a protein complex (cAb-protein [IL-2]-FITC labeled dAb) with cAb while incubated for 20 min at 4 °C. Later, the assay chamber was washed 2–3 times using PBS containing Tween 20, and the fluorescence signal from the protein–antibody conjugate was measured using a fluorescence microscope (IX-73; Olympus, Tokyo, Japan) with a filter unit (Cy5-4040C, Olympus) and CMOS camera (ORCA-Flash 4.0; Hamamatsu Photonics, Hamamatsu, Japan). Exposure to UV light with 495 nm wavelength was provided during measurement. The IL-2 concentration was determined from the fluorescence signal.

## Results and discussion

### Device design, pumping, and solution exchange

The solution exchange for immunoassay necessitates introducing the desired solutions into the assay chamber without entering the other μFCs, and successive removal through the exit port. To this end, we first constructed a type 1 microfluidic device with seven 200 μm wide inlet flow channels and an outlet flow channel that surrounded a reaction chamber of 2 mm diameter (Fig. S1A†). Each channel was constricted with a 50 μm-wide narrow structure at the entry to the reaction chamber, and each contained an inlet port and a solution reservoir. Narrowing the PDMS μFC to ∼50 μm can provide a hydrophobic barrier;^27,32^ therefore, we can prevent the undesired delivery of multiple solutions in the reaction chamber and through other μFCs (Fig. S4†).

Using the MRP, we successfully transported solutions to the reaction chamber without them entering other channels (Fig. S5A, B†). Nonetheless, we experienced problems with solution removal due to the imbalance of suction pressure and positions of the inlet channels, particularly those situated near the outlets. The solutions were withdrawn improperly, leaving undesired residues in the reaction chamber (Fig. S5C†) and the inlet μFCs (Fig. S5C, D†). This could contaminate other solutions and was not expected for solution exchange.

**Fig. 5:**
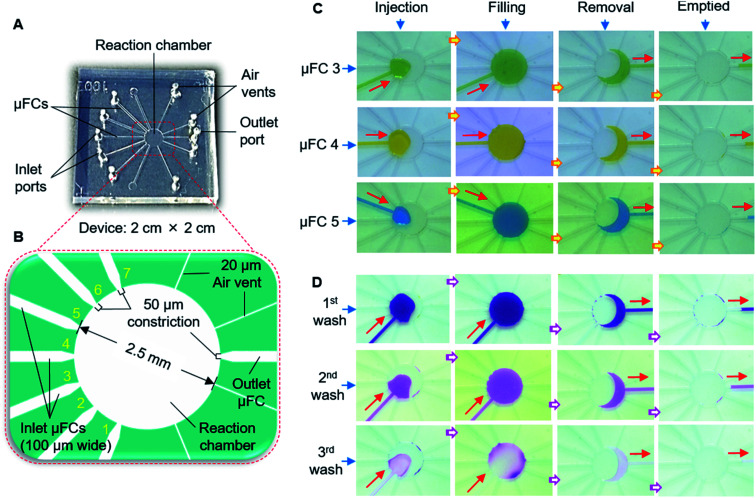
Valve-free scheme for sequential exchanging of multiple solutions. (A) Photograph of the type 2 microfluidic device. (B) Magnified view of the device showing the locations of the 50 μm constrictions and dimensions of seven μFCs, a reaction chamber, four air vents and a common outlet. The yellow numbers denote the positions of micro-flow channels (μFCs). (C) Images presenting sequential exchange of several (3 of 7) solutions. The steps involves serial injection, filling and removal of solutions. (D) Sequential solution exchange using single μFC with washing steps required for the bioassay. The red arrows indicate the direction of solution flow.

To solve the problems, we fabricated the type 2 device (Fig. 5A, B; S1B†). The width of μFCs was narrowed to 100 μm with the 50 μm constriction at the front. The inlet and outlet μFCs were placed at opposite sides of the reaction chamber (2.5 mm diameter), and the solution reservoirs in the middle of the inlet μFCs of the type 1 device were removed. Air vents (of 20 μm width) were additionally formed near the outlet to facilitate the proper injection and filling of solutions without air trapping. With the type 2 device, we succeeded in delivering and maintaining the solutions in the reaction chamber without penetrating the other μFCs, facilitating practical sequential solution exchange. Fig. 5C demonstrates the stepwise process of sequential injection, filling, and subsequent removal of seven dye solutions when employing the device. None of the solutions moved to other μFCs or fragmented during the exchange process. No residue was left in the reaction chamber while smoothly pumping out the solutions using suction. This simple, new microfluidic scheme restrained the solutions in the reaction compartment for over 30 min. In immunoassay systems, frequent washing of the residual solution from the assay area is essential for producing an accurate signal. In this simple device, a single μFC can be effectively engaged to perform multiple washes without any interruption (Fig. 5D). Pumping in the long soft tube is subjected to extra back pressure because of the higher tube wall friction. Furthermore, the soft tube assists in fluidic pulsation absorption.^19^ The MRP also carries those features in its operation. As a consequence, MRP delivered a smooth pumping experience while exchanging solutions in the reaction chamber.

The solutions were transported and withdrawn to and from the reaction chamber slowly (at a flow rate of ∼6 μL min^−1^) using the MRP. We calculated the flow rate based on the ratio of solution volume transported and the time required to fill and empty the reaction chamber by the MRP action. With the MRP, we tested and succeeded in transporting solution volume over 30 μL in the tube. As the pump is manually operated, it was not easy to achieve a constant flow rate. However, the MRP can be used whenever an operator needs it.

**Fig. 6:**
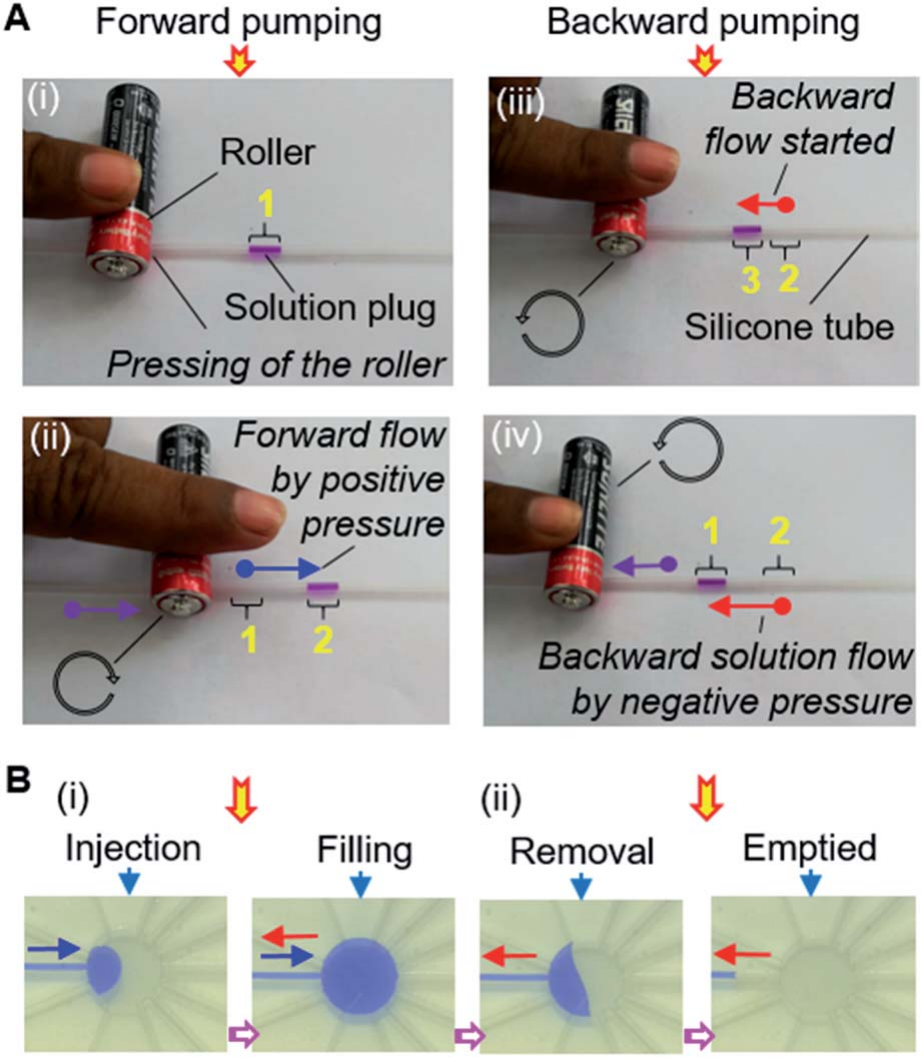
Bidirectional MRP. (A) Operation of MRP for the bidirectional pumping in silicone tube. (i and ii) Forward pumping for frontward solution delivery. (iii and iv) Backward pumping for backflow of solution. (B) Demonstration of bidirectional pumping in microfluidic device. (i) Solution injection and filling using forward pumping. (ii) Solution removal and returning to the inlet reservoir involving backward pumping. The blue and red arrows show the direction of forward and backward flow of the solution plug respectively; whereas, black (circular) and purple arrows indicate the rolling direction and displacement of the roller. Yellow numbers point towards the positions of the solution plug during operation.

Interestingly, MRP can enable bidirectional pumping. The bidirectional motion of the roller from the same side of the solution plug correspondingly can initiate the forward and backward flow of a solution by utilizing the positive and negative pressure of the MRP (Fig. 6A). Consequently, solution exchange without outlet μFCs can be accomplished in a microfluidic device using the bidirectional MRP (Fig. 6B).

While performing immunoassays, the surface of the substrate can be hydrophobic, leading to becoming air trapped inside the chamber by non-specific or physical adsorption of the antibody, antigen, or other biomolecules. The presence of air in the assay chamber may produce a misleading signal by altering the antibody immobilization and binding process because air can deteriorate the quality of proteins and cause light scattering.^33^ Therefore, we examined the effect of hydrophobicity on solution exchange using the devices formed with an intentionally formed hydrophobic layer of negative photoresists (*i.e.*, SU-8-25 and OMR-83) or PDMS beneath the reaction chamber. The contact angle of the water (1 μL) for PDMS-stamped, SU-8-25, and OMR-83 surfaces were 59°, 73.8°, and 90°, respectively (Fig. S6A†), and no problems with solution transport, filling, and withdrawal was observed due to the increase of hydrophobicity (Fig. S6B†).

Finally, we constructed the type 3 device (Fig. 2A; S1C†), with slight modifications to the design of the type 2 device, to make it simpler and reduce the reaction volume of the assay. The number of inlet μFCs was reduced to five, and the width of all inlet μFCs was made 50 μm along their entire length instead of forming definitive constriction, and the assay chamber was downsized to 1.5 mm. Air vents were widened to 30 μm and placed closer to an outlet with 200 μm width and 50 μm constriction. The reaction chamber effectively enclosed the solution, preventing penetration to any μFC (Fig. 2B). The device was employed for exchanging assay reagents in the immunoassay mentioned later.

BSA is a popular blocking reagent applied in immunoassays to prevent the undesired adsorptions of biomaterials;^41,47^ nonetheless, the BSA concentration should be adjusted to enable smooth transport and filling of solutions in the assay chamber without generating air bubbles. We tested the feasibility of exchanging protein solutions in the type 3 device with BSA solutions of 0.1–1% concentration. The device presented no issues of bubble trapping during the solution exchange operation for BSA solutions up to 0.5%. In contrast, concentrations above 0.5% sometimes resulted in the air becoming trapped inside the reaction chamber (Fig. S7†). Accordingly, a 0.5% BSA solution was chosen for blocking.

### Immunoassay applications

**Fig. 7:**
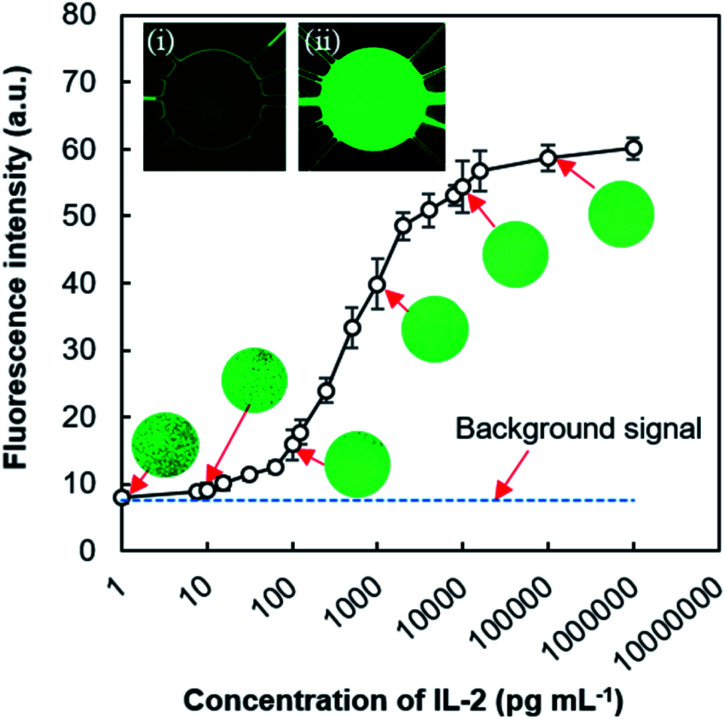
Fluorescence signal from on-device immunoassay showing the reliance on IL-2 concentration. Some exemplary fluorescence microscopy images are positioned near the corresponding values. Images are 100% zoomed under 10× magnification lens. Inset shows the response of devices with (i) blank and (ii) 1 μL mL^−1^ antigen samples. Error bars in graph signify the standard deviation for three replications.

To show the practicality of the MRP and valve-free device, we performed a sandwich fluorescence immunoassay for human IL-2 after the compatibility testing of MRP-driven exchange of assay reagents in type 3 device (ESI and Fig. S8†). For the immunoassay purpose, inlet μFC 3 was used to transport the coating buffer mixture, APTES (1%, v/v), and cAb. μFCs 2 and 4 were used to deliver the blocking reagent (0.5% BSA + 0.01% Tween 20) and the mixture of FITC-labeled dAb and antigen (IL-2), respectively. μFCs 1 and 5 were used for washing. For ELISA, either μFC 1 or 5 can be used to transport either the substrate or washing solution. The fluorescence signal derived from the formed protein complexes (*i.e.*, cAb-Ag-FITC-labeled dAb) was measured only from the reaction chamber. Fig. 7 shows that fluorescence intensity depends on IL-2 concentration. The fluorescence image taken from the reaction chamber indicated the variation in intensity due to the change of IL-2 concentration, corresponding to the amount of IL-2 trapped by the immobilized cAb. Some faint fluorescence was also observed for the blank sample without IL-2, which indicated the non-specific adsorption of FITC-labeled dAb onto the surfaces of the assay chamber. The intensity increased with the increment of IL-2 concentration in the range of 125 pg mL^−1^ to 2.0 ng mL^−1^ and inclined to saturate after that range. The platform showed the limit of detection (LOD) for IL-2 as 80 pg mL^−1^, the average response value for blank and three times its standard deviation. The LOD of our device lies within the normal range of IL-2 concentrations (*i.e.*, 10 pg mL^−1^ to 1 ng mL^−1^) in serum.^42^

Recent research has explored the elevated levels of IL-2 found in mild or asymptomatic COVID-19 cases and highlighted the latent use of IL-2 among cytokines as prognostic biomarkers for pandemic SARS-CoV-2 infection and degree of immunity after vaccination.^43,44^ Though our current LOD does not satisfy the required level (below 10 pg mL^−1^) of IL-2 detection to diagnose COVID-19 infection; an improved suitable nanomaterial-assisted assay^15^ could meet that diagnostic demand.

**Fig. 8:**
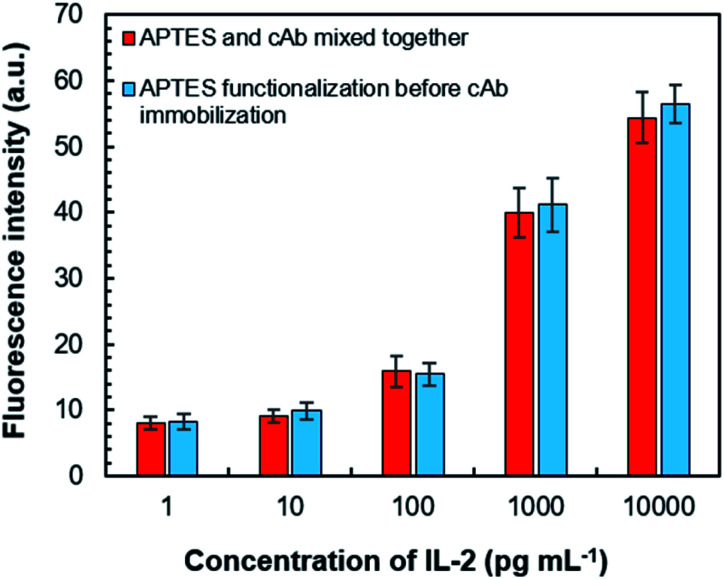
Comparative performance of two APTES-based cAb immobilization methods. Error bars in graph symbolize the standard deviation for three replications. APTES, (3-aminopropyl) triethoxysilane; cAb, capture antibody.

We also investigated the usefulness of APTES-based cAb immobilization. This was followed up by two methods of immobilization for the assay chamber—the first one was by delivering the freshly mixed solution of coating buffer, APTES (1% v/v) and cAb; the second one was functionalization with APTES solution (for ∼25 min) followed by the injection of a mixed solution of cAb and coating buffer. For both approaches, they were incubated for 25 min because APTES-based cAb immobilization over this period can result in a maximum signal response in the assay process, which can be stabilized for up to 7 days.^33^ Both the methods were found effective for cAb immobilization based on the similar fluorescence responses from the assay chamber (Fig. 8). However, the former decreased the number of operational steps; therefore, it was adopted for our study.

Later, we studied the surface blocking effect of BSA to prevent non-specific adsorption of FITC-labeled dAb (Details in ESI†). The 0.5% BSA by itself and together with a surfactant (0.01% Tween 20) can reduce the noises of non-specific protein adsorptions by 35% and 55%, respectively, when compared to an unblocked surface (Fig. S9†). The considerable reduction in non-specific protein adsorption by adding the surfactant with BSA indicates the addition of a hydrophilic property (by providing a hydroxyl group) to the hydrophobic wall of PDMS lessens the hydrophobic interactions that cause undesirable binding of proteins with the surfaces.^48^

The total time needed for our immunoassay was approximately 80 min including the time for the immobilization of capture antibodies and blocking with BSA, which is much shorter than the traditional ELISA.^12^ If the reaction chamber is already functionalized with cAb and blocked with BSA, the assay time is shortened further to approximately 20 min, which will be acceptable in emergencies or resource-deficient regions.

### Scope of MRP integration

The MRP is favorable for integrating microfluidic systems where stopped-flow solutions,^20,35^ a liquid plug,^25,32,34^ or column^33^ are necessary for bioassays. Furthermore, the MRP can process multiple liquid plugs (Fig. S10†), which can suit the system that requires the transport of a series of liquid plugs.^32,34^ The bidirectional MRP is well suited for platforms where the backward flow of the solutions is necessary.^25,34^ The ESI and Fig. S11† shows a conceivable microfluidic solution exchange scheme that utilizes the bidirectional MRP for bioassays. In this case, inlet chambers or tubes will act as waste reservoirs where forward pumped solutions can be retrieved with MRP-driven backflow from the assay site to the inlet reservoirs after solution processing (*i.e.*, immobilization/functionalization, assay reactions, and washings followed by incubation). MRP can also actuate membrane displacement-based valves usually driven by the air pressure of pneumatic or peristaltic pumps,^20,21^ which will further expand the possibility of novel microfluidic devices with high performance.

## Conclusion

We demonstrated an easy-to-apply microfluidic system for the sequential exchange of multiple solutions, which can be performed by untrained staff to detect proteins or biomarkers through a bioassay process. By adopting an uncomplicated roller pump and simplifying the design of a microfluidic reaction chamber by connecting it with narrow flow channels and air vents, we facilitated sequential solution exchange without any cross-contamination of solutions. The manual roller pump, which is simply a hollow silicone tube with a small cylindrical rigid roller, can be a cheap alternative pressure source to pressure-driven pneumatic or syringe pumps that rely on expensive and bulky external devices; thus, this method alleviates cost and portability issues. With this system, we performed a stepwise fluorescence immunoassay for detecting IL-2 in 20 min with a LOD of ∼80 pg mL^−1^. Considering the weeklong stability of the APTES-based cAb-immobilized surface, the prefabricated and functionalized device can be applied for on-the-spot POCT. Although we used a microscope and benchtop analyzer for assay signal readouts, integration of a smartphone-based analyzer^49^ can make this approach simpler and more suitable to on-the-spot or urgent biosensing applications. Moreover, colorimetric or electrochemical assays can also be planned with this easy-to-operate solution exchange platform, though further investigation is needed. Controlled solution processing is the prime issue for realizing stepwise assays in disposable devices, which can be addressed by the MRP and valve-free micro-flow structures. Thus, our streamlined microfluidic scheme holds the potential to be applied for instantaneous bioassays or POCTs in emergencies such as pandemics or in resource-inadequate settings where many tests are necessary and trained manpower or materials are unavailable.

## Author contributions

Conceptualization: G. C. B., H. S.; project administration: G. C. B., H. S.; methodology: G. C. B., H. S.; data curation: G. C. B., H. S.; formal analysis: G. C. B., H. S.; investigation: G. C. B.; visualization: G. C. B.; validation: H. S.; writing-original draft: G. C. B.; writing-review and editing: G. C. B., H. S.; funding acquisition: G. C. B., H. S.

## Conflicts of interest

There are no conflicts of interest to declare.

## Supplementary Material

RA-012-D1RA08052K-s001
