# Supplementary material for: Simple manual roller pump-driven valve-free microfluidic solution exchange system for urgent bioassay
Source: RSC Adv. 2022 Jan 21;12(5):2938–46. doi: 10.1039/d1ra08052k (PMC8979114; doi:10.1039/d1ra08052k)
Supplement: RA-012-D1RA08052K-s001 [file RA-012-D1RA08052K-s001.pdf]

Electronic Supplementary Material (ESI) for RSC Advances.
This journal is © The Royal Society of Chemistry 2022

Electronic Supplementary Information

Simple manual roller pump-driven valve-free microfluidic solution exchange system for urgent bioassay

Gokul Chandra Biswas*[a] and Hiroaki Suzuki[b]

[a] *School of Life Sciences, Shahjalal University of Science and Technology, Sylhet-3114, Bangladesh*

[b] *Graduate School of Pure and Applied Science, University of Tsukuba, Tsukuba, Japan.*

**Email: gcbiswas-geb@sust.edu*

SU-8 molding for patterning in PDMS substrate

The glass wafer was first cleaned by immersion in a boiled solution mixture of ultra-pure water (H_2O), 25% NH_3, and 30% H_2O_2 in a volumetric ratio of 4:1:1 for 5 min and followed by several washes in boiled distilled water. The glass wafer spin-coated with the SU-8 25 photoresist was prebaked at 65 °C and 95 °C for 10 min and 45 min, respectively, using a hot plate. Afterward, the SU-8 25 substrate was exposed to UV light through a specific photomask, and postbaked at 65 °C and 95 °C for 5 min and 10 min, respectively, to develop the SU-8 25 photoresist and generate a mold of specific structures. The immediate mixture of a PDMS prepolymer solution and a curing agent was decanted into the mold and was cured for 30 min at 80 °C in an oven. After cooling, the PDMS was peeled off from the mold and cut into the proper sizes of the chips.

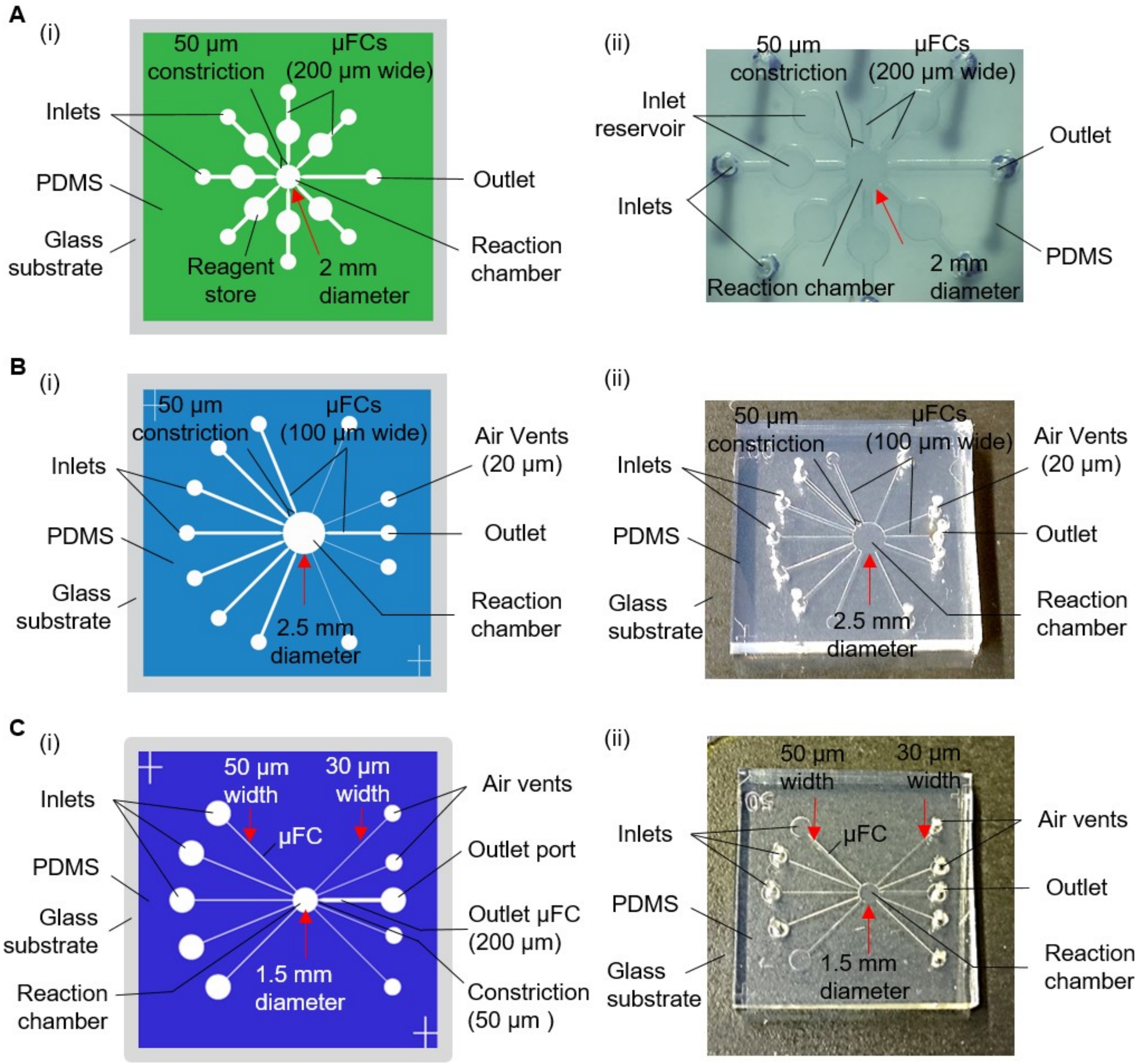

Fig. S1 Microfluidic devices used for solution exchange showing positions and dimensions of the micro-flow structures. (A) Type 1 device used for primary experimentation of solution exchange. (B) Type 2 device exploited for successful demonstration of solution exchange. (C) Type 3 device employed for reagent exchange in immunoassay. (i) and (ii) under (A-C) indicate the illustrated top view of the devices and the top snap-shot of the fabricated devices, respectively. The manual roller pump (MRP) is operated to process the solutions in all devices. µFC, micro-flow channel.

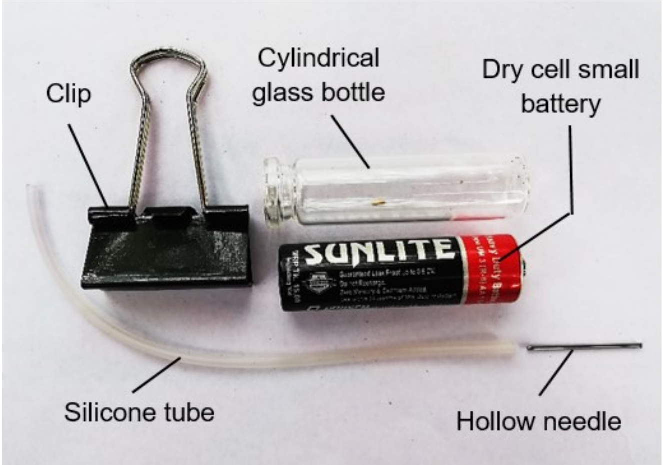

Fig. S2 Materials used for the manual roller pump. The silicone tube is for containing air and solution. The cylindrical glass bottle or dry cell battery act as rollers. The needle connects the silicone tube and inlet port of the microfluidic device. Clip is used for clamping the silicone tube, if necessary.

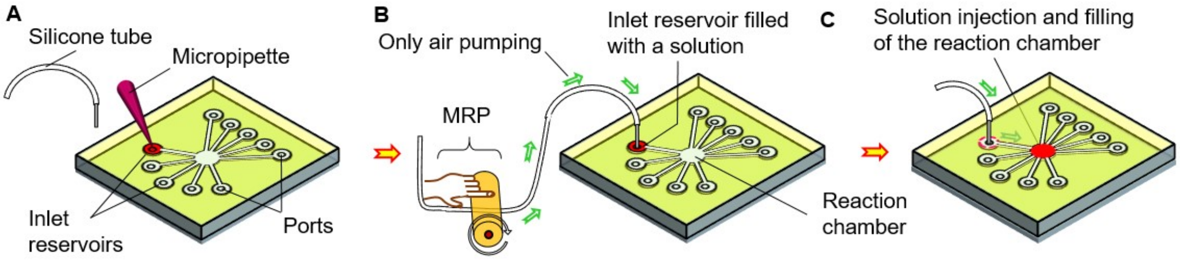

Fig. S3 Solution processing using MRP-based air movement in the type 3 device. (A) A solution is first loaded into the inlet reservoir with a micropipette. (B) The MRP is connected to pump air by rolling only. (C) The solution is transported to the reaction chamber utilizing the pumped air from MRP.

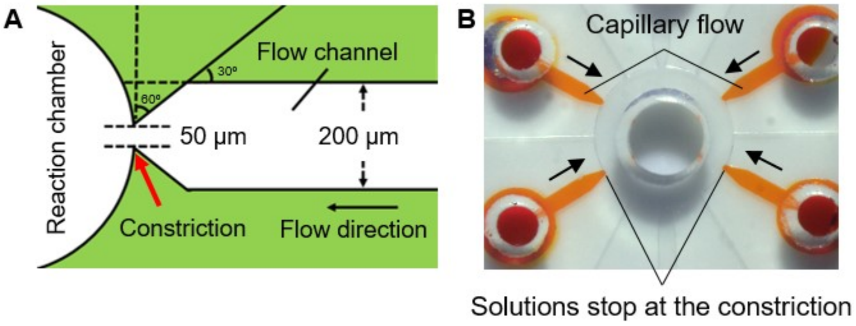

Fig. S4 (A) Outline of the 50-µm constriction in the µFC at the entry to the reaction compartment. (B) Image displaying the stoppage of capillary solution flow by the 50-µm narrow structure.

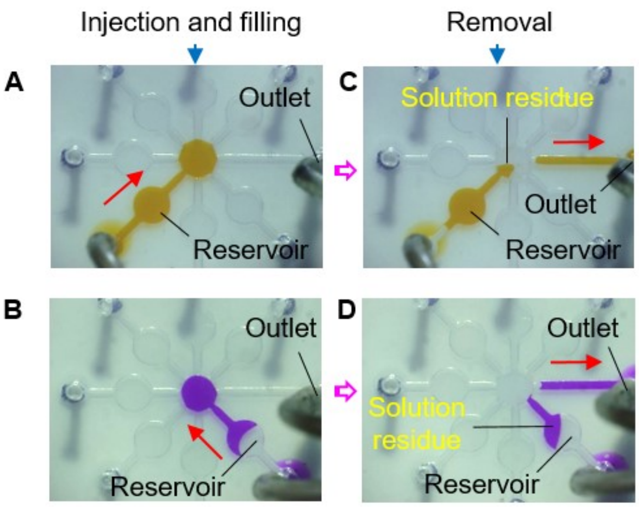

Fig. S5 Improper solution removal in type 1 microfluidic device. (A) and (B) Proper solution injection and filling in the reaction chamber without penetrating other μFCs regardless of their positions. (C) Improper removal making parted solution residue in the reaction chamber due to the imbalance in negative pressure against the volume in the reservoir. (D) Incomplete removal parting the solution at the entrance of the μFC due to the positional unsuitability of the inlet μFC. The red arrows indicate the flow direction of solutions.

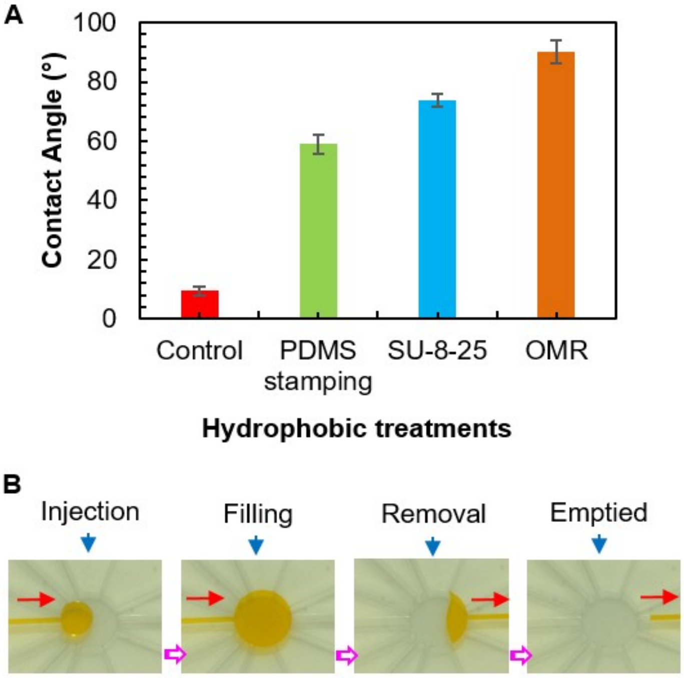

Fig. S6 Effect of hydrophobicity on solution flow. (A) Change in contact angle on artificial hydrophobic surfaces of polydimethylsiloxane (PDMS) stamping, and SU-8-25 and OMR photoresists coated on the glass substrate. (B) Smooth solution processing (i.e., injection, filling, and removal) on the OMR-coated hydrophobic surface. Error bars represent the standard deviation of five replications.

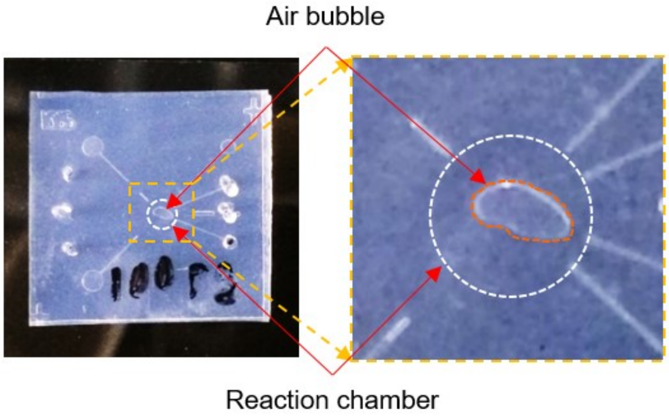

Fig. S7 Trapping of air bubbles (enclosed with red dot line) inside the reaction chamber (white circle with dotted line) for >0.5% BSA concentration.

Compatibility testing of MRP and type 3 device for bioassay

We tested the compatibility of MRP for the exchange of assay reagents (except cAb) in a type 3 device targeting a bioassay. The assay chamber was functionalized with a mixture of coating buffer, APTES (1 % v/v), FITC-labeled dAb, followed by surface blocking with 0.1 % BSA and three times washing. Each assay solution was exchanged by MRP and incubated for 25 min except for washing buffer. The fluorescence signals occupied a substantial area in the reaction chamber (Fig. S8), which proved the potential applicability of the MRP and type 3 device in a joint venture for effectual functionalization of the reaction chamber required to run an immunoassay.

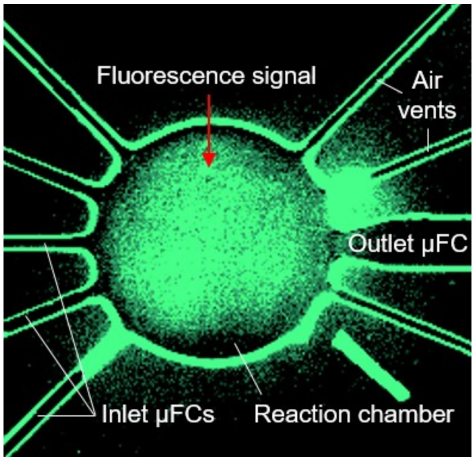

Fig. S8 Suitability testing of the proposed MRP and type 3 microfluidic system for bioassay. The image shows the substantial amount of fluorescence signal occupancy in the reaction chamber after MRP-based exchange of assay reagents (except capture antibody). μFC, micro-flow channel.

Effect of surface blocking with BSA and Tween 20

Three different treatment conditions of the assay chamber – (i) unblocked, (ii) blocked with 0.5% BSA, and (iii) blocked with 0.5% BSA plus surfactant (0.01% Tween 20) for 30 min were adopted to study the effect of surface blocking with BSA and surfactant. The treatments were applied after rinsing of the assay surface with ethanol and water followed by (3-aminopropyl)triethoxysilane (APTES)-based cAb immobilization. The fluorescence signal from the incubation of the FITC-labeled dAb in the assay chamber indicated the state of protein adsorption upon different surface treatment conditions (Fig. S9). The unblocked surface was more vulnerable to the non-specific protein adsorption than the blocked surfaces.

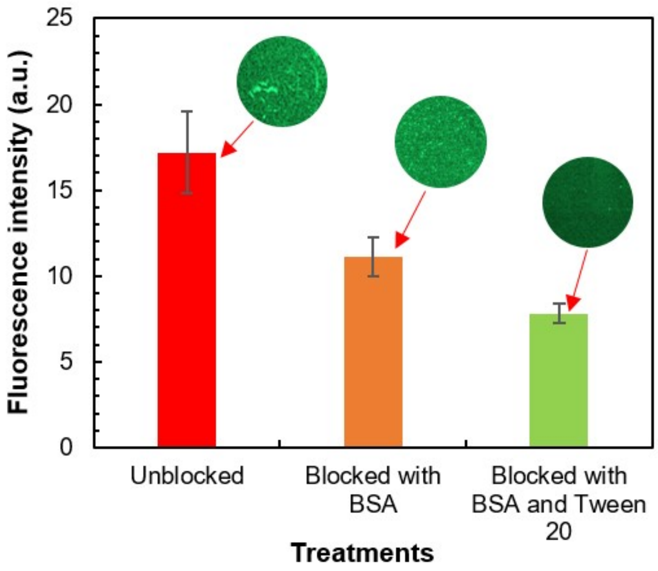

Fig. S9 Comparative responses of surface treatments for avoiding non-specific adsorption of FITC-labeled dAb in the reaction chamber. The treatments are- (i) unblocking, (ii) blocking with 0.5% BSA and (iii) blocking with 0.5% BSA and Tween 20. The fluorescence microscopy images in the inset correlate to the respective treatments. Error bars show the standard deviations of three replications.

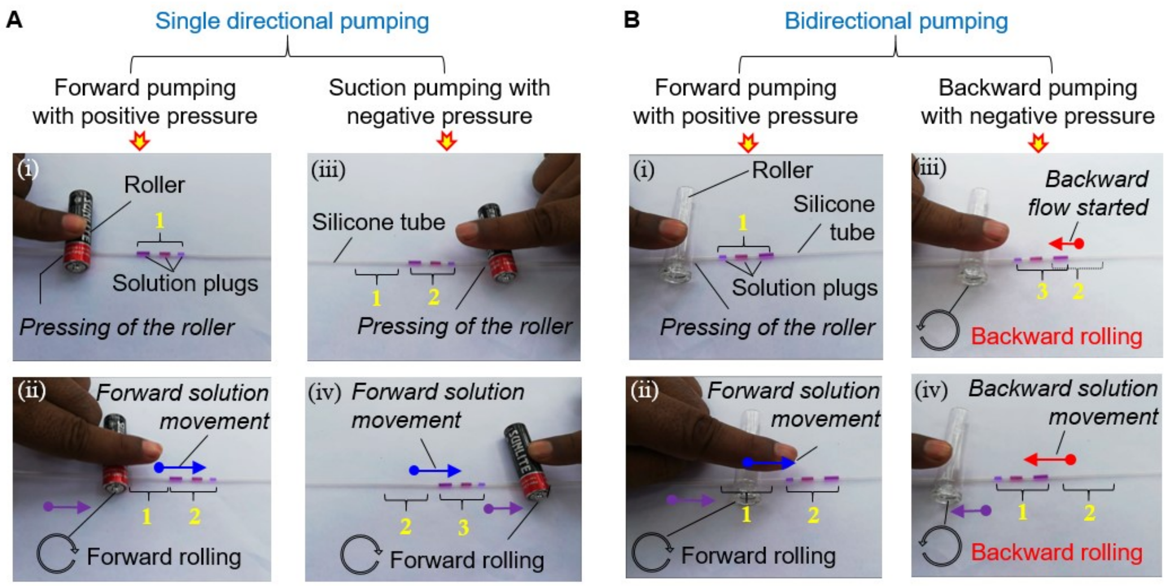

Fig. S10 Multiple solution processing by MRP. (A) Operation of MRP for the single directional pumping of multiple solution plugs in the silicone tube. (i and ii) Forward and (iii and iv) suction pumping with forward rolling of the roller for forward solution movement. (B) Bidirectional pumping of multiple solution plugs with the MRP. (i and ii) Forward and (iii and iv) backward pumping with forward and backward rolling of the roller for forward and backward solution movement. The blue and red arrows show the direction of forward and backward flow of the solution plug, respectively; likewise, black (circular) and purple arrows indicate the rolling direction and displacement of the roller. Yellow numbers point towards the positions of the solution plug during operation.

MRP-based bidirectional pumping for bioassay

Fig. S11 represents the scheme of MRP-based bidirectional pumping for solution exchange in microfluidic device usable for the bioassay. Each of the inlet reservoirs and flow channels is assumed to contain the individual or mixed reagents (such as capture antibody, APTES, coating buffer, detection antibody, washing buffer, labeling material, or others) necessary for bioassay and process respectively for accomplishing a targeted bioassay. Solutions from the inlet reservoir are sequentially pumped into the reaction chamber for the desired reaction or washing using forward MRP; whereas, the processed solutions are returned to inlet reservoir using backward MRP. Thus, the solution exchange system, extra processing steps for outlet will not be required. This model contains eight flow channels. However, the number of flow channels can be adjusted according to the number of reagent solutions to be processed for bioassay.

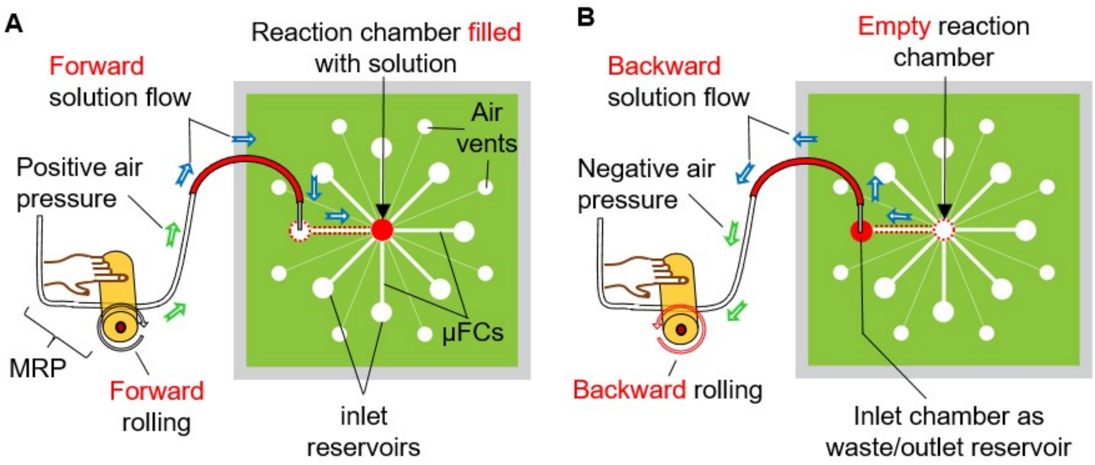

Fig. S11 Layout of a simple microfluidic solution exchange scheme using MRP-based bidirectional pumping for bioassay. (A) Solution insertion from inlet reservoir/tube to the reaction chamber by forward pumping (i.e., forward MRP operation). (B) Solution removal by backward pumping (i.e., backward MRP operation) from the reaction chamber to the inlet reservoir/tube that serves as waste/outlet reservoir. The green and blue arrows indicate the direction of air and solution movement respectively.
